# Nephronectin promotes cardiac repair post myocardial infarction via activating EGFR/JAK2/STAT3 pathway

**DOI:** 10.7150/ijms.71780

**Published:** 2022-05-13

**Authors:** Yaping Zhang, Di Wang, Zhe Zhao, Liang Liu, Guofang Xia, Tianbao Ye, Yu Chen, Congfeng Xu, Xian Jin, Chengxing Shen

**Affiliations:** 1 Department of Cardiology, Shanghai Jiao Tong University Affiliated Sixth People's Hospital, Shanghai 200233, China; 2 Department of Geriatrics, Shanghai Jiao Tong University Affiliated Sixth People's Hospital, Shanghai 200233, China

**Keywords:** Nephronectin, myocardial infarction, epidermal growth factor receptor (EGFR), angiogenesis, cardiac repair

## Abstract

**Background**: ECM proteins are instrumental for angiogenesis, which plays momentous roles during development and repair in various organs, including post cardiac insult. After a screening based on an open access RNA-seq database, we identified Nephronectin (NPNT), an extracellular protein, might be involved in cardiac repair post myocardial infarction (MI). However, the specific impact of nephronectin during cardiac repair in MI remains elusive.

**Methods and Results**: In the present study, we established a system overexpressing NPNT locally in mouse heart by utilizing a recombinant adeno-associated virus. One-to-four weeks post MI induction, we observed improved cardiac function, limited infarct size, alleviated cardiac fibrosis, with promoted angiogenesis in infarct border zone in NPNT overexpressed mice. And NPNT treatment enhanced human umbilical vascular endothelial cell (HUVEC) migration and tube formation, putatively through advocating phosphorylation of EGFR/JAK2/STAT3. The migration and capillary-like tube formation events could be readily revoked by EGFR or STAT3 inhibition. Notably, phosphorylation of EGFR, JAK2 and STAT3 were markedly upregulated in AAV2/9-cTnT-NPNT-treated mice with MI.

**Conclusions**: Our study thus identifies the beneficial effects of NPNT on angiogenesis and cardiac repair post MI by enhancing the EGFR/JAK2/STAT3 signaling pathway, implying the potential therapeutic application of NPNT on myocardial dysfunction post MI.

## Introduction

Despite dramatic therapeutic advancements, cardiovascular disease (CVD) remains the leading threat to human health worldwide in recent decades^1^, ^2^. Myocardial infarction (MI), one of the most notorious CVD, usually gives rise to irreversible tissue damage, and elicits pathological ventricular remodelling and heart failure eventually. An array of studies have been focused on preserving cardiomyocytes^3^, ^4^, increasing angiogenesis^5^, and ameliorating detrimental fibrotic remodelling^6^, ^7^. After MI, formation of new blood vessels restores oxygen and nutrient supply to the infarcted tissue and is deemed to be a key factor for adequate post-ischemic repair^8^, ^9^. Apart from the capacity to salvage ischemic myocardium at early stages after MI, proangiogenic therapy is also indispensable for long-term left ventricular remodelling to prevent the transition to heart failure^9^-^11^. Hence, much effort has been invested to unearth effective proangiogenic factors.

The extracellular matrix (ECM) is the non-cellular component present within all tissues and organs. The historical notion of ECM as static and inert scaffold providing physical support for the cellular constituents has been challenged by the mounting evidence concerning ECM modulating cellular phenotype and function, including cell proliferation, differentiation, and survival^12^, ^13^. Furthermore, a growing body of evidences suggest that ECM alterations play a role in orchestrating fibrosis, angiogenesis, and regeneration in ischemic hearts^14^, ^15^. In this context, targeting the ECM may mediate angiogenesis and benefit repair after MI. Based on an open access RNA-seq data at Gene Expression Omnibus concerning multicellular transcriptional analysis in infarcted and noninfarcted neonatal (P1) and adult (P56) mouse hearts, we identified nephronectin (NPNT), a 70-90 kDa extracellular matrix protein, as a potential candidate involving in cardiac injury and repair. Then we established a cardiac-specific NPNT overexpressing system, and further explored the potential role and related mechanisms, and we found that NPNT enhance angiogenesis, attenuate remodelling, and improve survival eventually, shed a light on potential therapeutic application of NPNT on myocardial repair post MI.

## Methods

### Animals

Eight- to ten-week-old C57BL/6 male mice were obtained from Slac Laboratory Animal Co., Ltd. (Shanghai, China). All mice were housed under standard conditions of lighting (12/12 h light/dark cycles) and temperature (22-24°C) throughout the experiment. Experiments were performed under a project license (No.: DWLL2022-0014) granted by the Ethics Committee of Shanghai Jiao Tong University Affiliated Sixth People's Hospital, in compliance with the National Laboratory Animal Management Regulations and guidelines for the care and use of animals.

To overexpress NPNT in MI mouse hearts, recombinant adeno-associated virus (serotype 2/9) vectors carrying mouse NPNT with a c-TnT promoter (AAV2/9-cTnT-NPNT-GFP) were used. AAV2/9-cTnT-GFP served as a negative control (GENE Inc., Shanghai, China). Four weeks before the establishment of MI, 50 µl of PBS and 50 µl of solution containing adeno-associated virus NPNT-GFP (1.0×10^12^ PFU/mL) and EV-GFP (1.0×10^12^ PFU/mL, EV-GFP: empty vector-green fluorescent protein) were injected into myocardium of left ventricle under echocardiographic guidance. Then, MI operation was induced by permanent left anterior descending coronary artery (LAD) ligation, as described previously^16^. Overall, 126 mice were randomly allocated to the sham group (n = 24), MI-PBS group (n = 30), MI-EV (n = 36), MI-NPNT (n = 36), respectively. 11 mice in total were excluded because of death during the MI operation (3 mice) or failure in MI model establishment via echocardiography test performed at day 1 post MI (8 mice). The mice used in the present study may be subjected for multiple purposes. For example, myocardial tissue used for Western blot can also be utilized in the immunohistochemistry staining.

### Echocardiography

On day 1 and day 28 post-MI induction, mice were anaesthetized with 2% isoflurane and their cardiac function was determined using a Visual Sonics Vevo 2100 system (Visual Sonics Vevo 2100). Values of ejection fraction (EF, %), fractional shortening (FS, %), left ventricular systolic internal diameter (LVID-s, mm) and left ventricular diastolic internal diameter (LVID-d, mm) were documented. Five consecutive cardiac cycles were checked and analysed. The operator was blinded to the allocation of animals.

### Assessment of histopathology

Mice hearts were harvested and embedded in paraffin as published before. Briefly, the ventricular tissues were cut to produce 4-μm sections from the apical to the basal part of the left ventricle. Masson trichrome staining were utilized to evaluate fibrosis area. Images were acquired using a Nikon microscope. Fibrosis area (Fibrosis area vs. cross section area, %) was calculated by Image J.

### Measurement of myocardial infarction size

On day 28 after the MI surgery, mice hearts from the four group (n=6 per group) were harvested immediately and cut into 2 mm sections to incubate with 1% tetrazolium chloride (TTC) for 10 min at 37℃, as previously described^17^. Infarct size was characterized by the pale regions and calculated using Image J.

### Cell culture

HUVEC and mBend3 (mouse brain endothelial cell) were maintained in Endothelial Cell Growth Medium (Gibco). Adult mouse cardiomyocytes were isolated from male C57BL/6 J mice via collagenase perfusion using a Langendorff-free method as previously described^18^.

### Plasmid construction

Mouse NPNT adenoviral plasmid was constructed using a gateway recombination system from Invitrogen. PLV-IRES-puro-NPNT was used as template to produce blunt end PCR products. The primer pairs were as follows: Forward: CACCATGGCTGTGCTCCTAGCG; Reverse: TCAGCAGCGACCTCTTTTCAAGCTC. PCR products were then purified with PCR purification kit (OMEGA, cat# D2500-02) and cloned into pENTR™/DTOPO® vector using pENTR™ Directional TOPO® Cloning Kits to generate an entry clone. The entry clone was then recombined to pAd/CMV/V5-DEST™ vector using LR to create adenoviral plasmid pAd/CMV/V5-DEST-NPNT. We identified the positive colonies by sequencing. pAd/CMV/V5-GW/lacZ was provided as control plasmid.

### Endothelial cell migration assay

A scratch wound-healing experiment could be detailed in previously published protocol^19^. Briefly, the cell monolayer was seeded in a 6-well plate, and a straight line was scraped with a p200 pipet tip to create a 'scratch'. Recombinant mouse NPNT (R&D, 500 ng/ml) or human bFGF (Pepro Tech, Inc., 50 ng/ml) protein were used in serum-free medium instead of complete medium. The cell migration images were taken at 0 hour and 12 hours. HUVEC and mBEND3 was preincubated with an EGFR inhibitor Gefitinib (MCE, Ca# HY-50895, 10 μM) or STAT3 inhibitor C188-9 (Selleck, Ca# S8605, 10 μM) for 1 hour before the wounding in the inhibitory assay.

Cell migration was also determined by a transwell assay by using a 8 μm pore-size polycarbonate membrane chamber (Corning)^20^. Briefly, 2×10^4^ cells were seeded into the upper chamber and 500 μl DMEM containing 10% FBS was added into the lower chamber. After 24-hour treatment with indicated proteins (rmNPNT, 500 ng/ml, bFGF, 50 ng/ml) or inhibitors (Gefitinib, 10 μM, C188-9, 10 μM) in the upper chamber, the cells were fixed with methanol for 30 min, followed by staining with 0.1% crystal violet for 15 min. The nonmigrating cells were wiped off gently and migrating cells were counted in 5 fields with a microscope.

### Endothelial tube formation assay

A tube formation assay was conducted as previously reported^20^. HUVECs were seeded onto a layer of Matrigel (BD, Ca# 356234) and cultured for 3 hours with serum-free medium containing recombinant mouse NPNT or human bFGF as a positive control. Quantification of branch points and tube length in 5 fields of vision for each was performed by Image J software (National Institutes of Health, USA).

### Western blot analysis

Western blots were performed as previously described^21^, and the following antibodies were used: anti-EGFR (1:1000, Ca# 4267), anti-p-EGFR (1:1000, Ca# 3777), anti-JAK2 (1:1000, Ca# 3230), anti-p-JAK2 (1:1000, Ca# 3776), anti-STAT3(1:1000, Ca# 9139), anti-p-STAT3(1:1000, Ca# 4093), anti-α-actinin(1:1000, Ca# 6487) (Cell Signalling Technology, USA). Antibody against NPNT was obtained from Abcam (1:1000, Ca# ab64419). Anti-EGFR neutralizing, clone LA1 (2 μg/mL, Ca# 05-101) was purchased from Sigma and EGFR dominant negative plasmid (pRK5RS-HERCD533) was obtained from Addgene.

### Quantitative RT-PCR

Total RNA was purified from frozen hearts using TRIzol reagent (Invitrogen), and 500 ng of RNA was reverse transcribed to cDNA using a 5×HiScript II Q RT SuperMix for qPCR (Vazyme, China). qRT-PCRs were performed using a 2×AceQ Universal SYBR qPCR Master Mix (Vazyme, China) according to the manufacturer's protocols. Fold change of gene expression was calculated using 2^-ΔΔCt^ method after normalized to a housekeeping gene (18S rRNA). Primer sequences are as follows:

NPNT-Forward, 5'-GAAGCCTCGGCCCTGTAAG-3', NPNT-Reverse, 5'-AGCATGTATCCGTTGAGACAGTA-3', 18S-Forward, 5'-AGTCCCTGCCCTTTGTACACA-3', 18S-Reverse, 5'-CGTTCCGAGGGCCTCACT-3'.

### Tissue immunofluorescent staining

Mice heart specimens were fixed and processed following our published protocols^21^. Briefly, the ventricular cryosections were cut to produce 10-μm sections from the apical to the basal part of the left ventricle. Antibodies used for immunofluorescent labelling were as follows: anti-CD31 (Novus, Ca# NB600-1475, 1:200), and anti-α-SMA (Abcam, Ca# ab5694, 1:1000). Images were acquired using a Nikon fluorescence microscope.

### RNA-seq data processing

RNA-seq data was obtained from Gene Expression Omnibus under the accession number GSE95755^22^. We screened significantly expressed extracellular matrix genes among indicated groups using Kyoto Encyclopedia of Genes and Genomes (KEGG) Pathway enrichment analysis and then generated the heatmap using Prism 7.0 (GraphPad Software, USA).

### Statistical analysis

All experiment data was analysed with Prism 7.0 (GraphPad Software, USA). Measurement data were presented as mean ± standard deviation. Unpaired two tailed *t-*tests were used for comparisons between two groups. A one-way analysis of variance with Tukey's post hoc test was applied when more than two groups were compared. In addition, log-rank testing was performed to determine statistical difference between multiple survival curves. Significance was considered with *P* value less than 0.05.

## Results

### Identification of NPNT and its function determination during cardiac repair in MI mice

**Figure 1:**
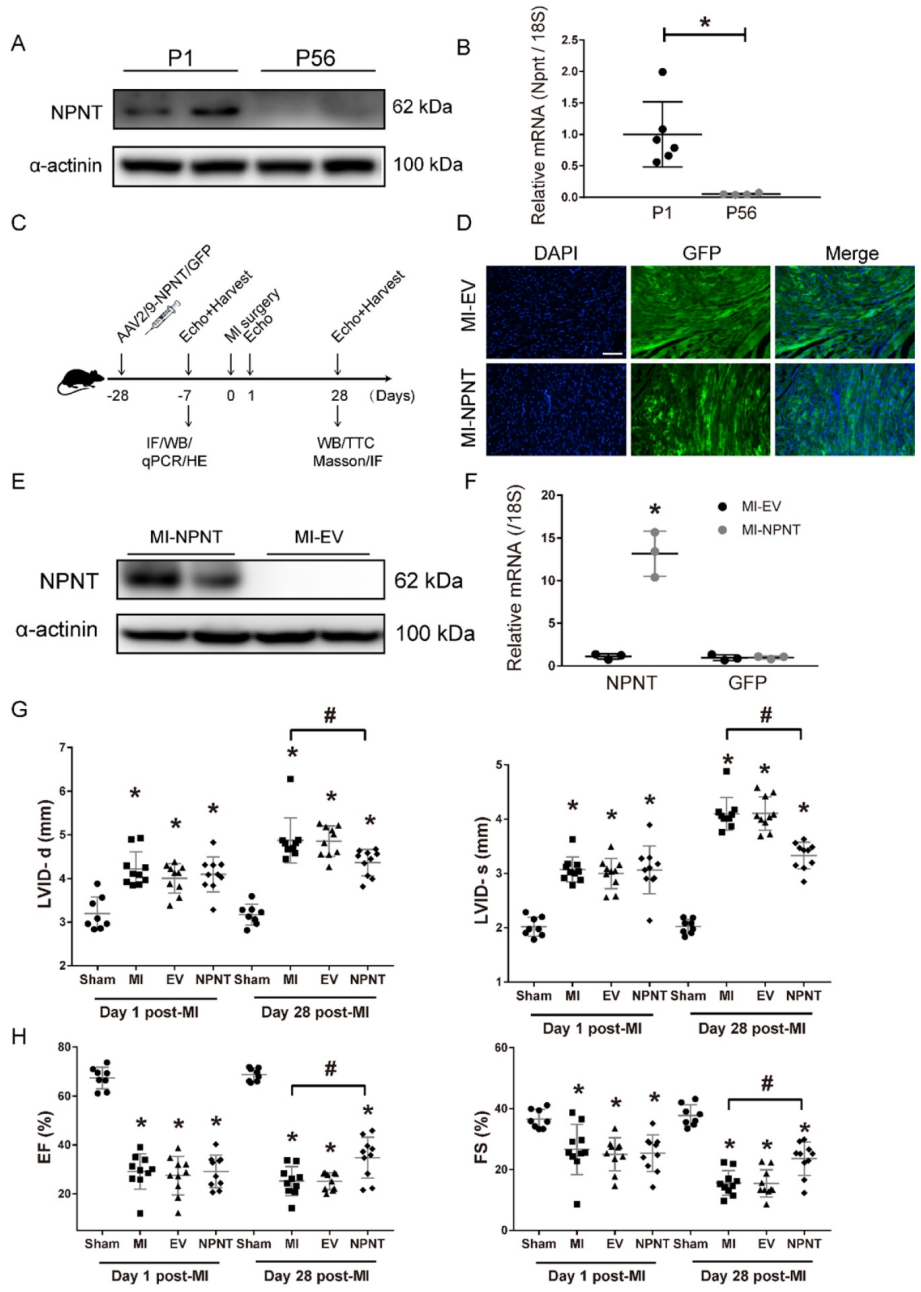
** NPNT gene overexpression by adeno-associated virus (serotype 2/9) in mouse hearts upregulated NPNT and attenuated cardiac dysfunction post-MI. (A-B)** NPNT was transiently expressed in mice heart both at protein and mRNA level. NPNT was expressed in P1 (neonate mice, n = 2 for WB, n = 6 for qPCR) but hardly detected in P56 (adult mice, n = 2 for WB, n = 4 for qPCR), * *P* < 0.05. **(C)** Protocol of AAV2/9-mediated NPNT overexpression and time points for tissue collection in adult male C57BL/6 mice. **(D)** GFP-expressing AAV was imaged in the myocardium (n = 2). **(E)**, **(F)** The protein and mRNA levels of NPNT in the infarct border zone were all significantly upregulated on 3 weeks post-injection (n = 3). * *P* < 0.05. Parameters of LVID-d, LVID-s **(G)**, EF and FS **(H)** detected on day 1 and day 28 showing that MI surgery caused equal decreased heart function among the three MI group. (n = 8-10). * *P* < 0.05 versus sham group, and # *P* < 0.05 versus MI-PBS group. Scale bar, 100 μm.

Previous studies demonstrated that in addition to the ability of maintaining correct matrix organization and function, extracellular matrix proteins are also essential for an array of biological processes, such as angiogenesis, via cellular communicating and interacting. Taking advantage of an open access on RNA sequencing database, we made a screening of ECM protein candidates involving in cardiac repair. Quaife-Ryan, et al^23^ isolated cardiomyocytes from infarct and noninfarct neonatal (1 day post-natal, P1) and adult (56 days post-natal, P56) mouse hearts at day 3 post surgery and then subjected to RNA sequencing to generate the transcriptome profile of the major cardiac cell population during cardiac development, repair, and regeneration. Utilizing bioinformatics analysis, we generated a heatmap (Supplementary Figure 1A) based on the expression of molecules classified as products of extracellular matrix genes in cardiomyocytes. Among these molecules, NPNT attracted our attention as a distinct difference gene between neonatal mice and adult mice. To explore the role of NPNT in cardiac development, we detected the protein expression and gene expression in neonatal mice and adult mice and confirmed that NPNT was transiently expressed in mice heart (Figure 1A, Figure 1B), implying a potential role in cardiac repair post MI injury.

To investigate the role of NPNT in adult mouse heart, we constructed a system with NPNT overexpressed on mouse heart specifically. In our system, the AAV2/9-NPNT-cTnT-GFP virus and a negative control were delivered into mouse hearts 28 days prior to MI surgery to overexpress the NPNT gene (Figure 1C). Three weeks post infection, several mice from the MI-EV and MI-NPNT group (n= 4 for each group) were executed to determine cardiac-targeted NPNT expression. Figure 1D showed green fluorescence protein was profoundly expressed in left ventricular myocardium. Moreover, the qPCR and WB results confirmed successfully overexpression of NPNT at both gene and protein levels in the MI-NPNT group compared with the control group (Figure 1E-1F). Of note, cardiac-targeted NPNT expression did not affect myocardial morphology, capillary density, and cardiac function based on HE staining (Supplementary Figure 2A), CD31 staining (Supplementary Figure 2B), and echocardiography (Supplementary Figure 2C-2E).

To assess cardiac function change, echocardiography was performed successively on day 1 and 28 post-surgery. As expected, LVID-s and LVID-d were expanded and values of EF, FS were correspondingly decreased in MI groups on day 1 post-MI, demonstrating significantly and equally exacerbated heart function. Notably, the NPNT-overexpressing mice showed ameliorated cardiac dysfunction 4 weeks post the surgery, evidenced by increased EF (34.8 ± 8.4% *vs.* 25.2 ± 5.9%,* P* < 0.05) and FS (23.6 ± 5.5% *vs.*15.6 ± 4.1%, *P* < 0.05) along with decreased LVID-d (4.4 ± 0.3 *vs.* 4.9 ± 0.5 mm, *P* < 0.05) and LVID-s (3.3 ± 0.3* vs.* 4.1 ± 0.3 mm, *P* < 0.05) versus mice in the MI-PBS group (Figure 1G-1H, Supplementary Figure 1B). These results indicate that NPNT overexpression attenuate heart dysfunction in MI mouse.

### NPNT overexpression limited infarct size, attenuated myocardial interstitial fibrosis, and benefited survival after MI

**Figure 2:**
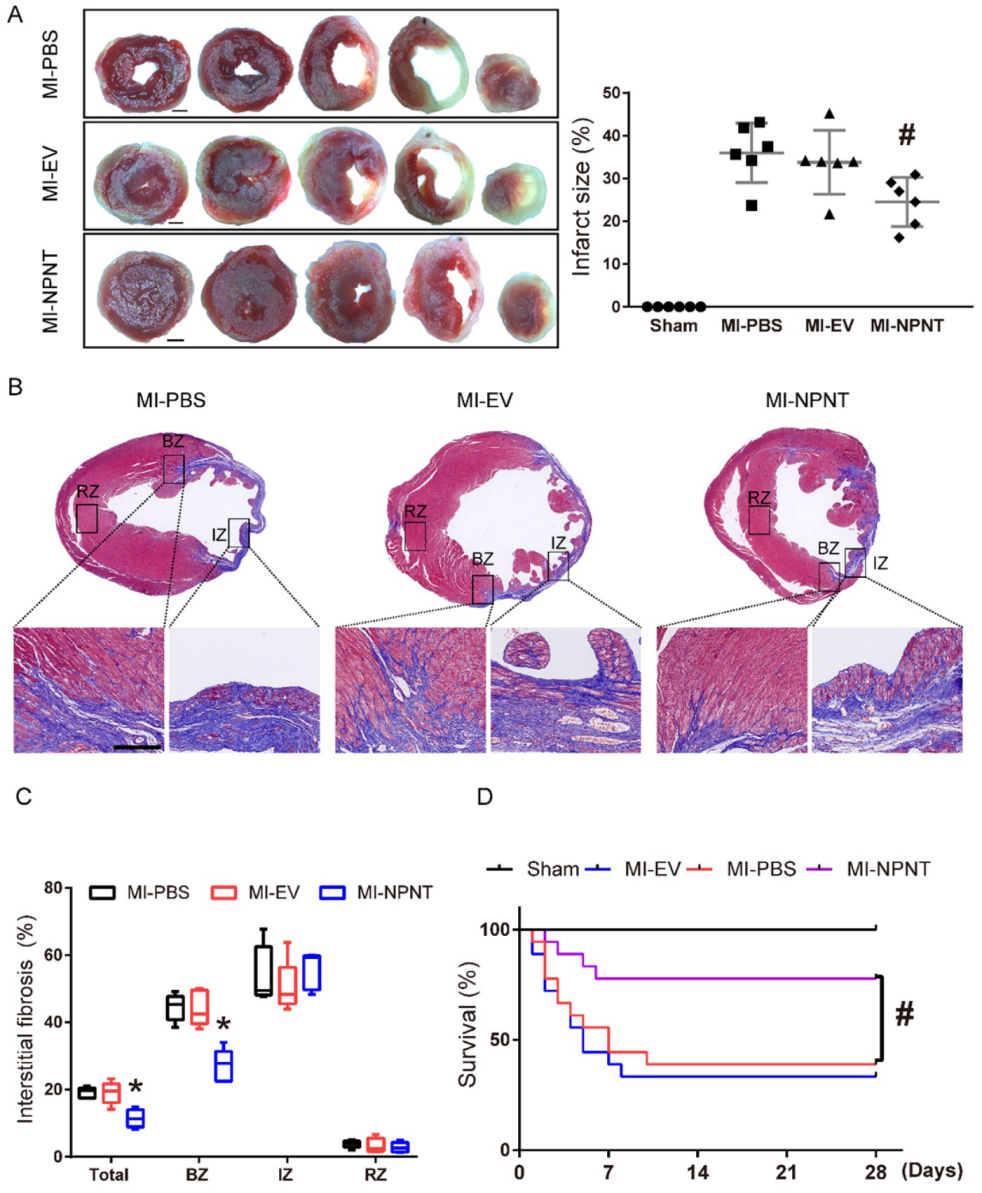
** NPNT limited myocardial infarction size, fibrosis, and improved survival after MI. (A)** Representative images of tetrazolium chloride staining at day 28 post-MI in the mice hearts from the four groups. Ratios of infarcted area vs. left ventricular tissue area are calculated in the graph (n = 6). Scale bar, 1 mm. **(B)** Masson trichrome staining of infarcted mouse myocardium in different groups on day 28 after MI within infarct zone, border zone, remote zone (n = 5). **(C)** Ratios of fibrosis area vs. total cross section area are analysed using Image J.** (D)** Survival analysis within 28 days showed significantly lower mortality in the MI-NPNT group compared with other two MI groups. (*P* = 0.0197) (n = 18). # *P* < 0.05 versus MI-PBS group. Scale bar, 100 μm.

Myocardial infarction may lead to cardiomyocytes death, excessive fibrils deposition and impaired survival. To further assess the effects of NPNT on MI sequelae, TTC staining was employed on day 28 post-MI to detect myocardial infarction size, and we also examined interstitial fibrosis along with survival analysis on day 28 post-MI. Figure 2A showed the representative TTC staining. As shown in the result, infarct size in NPNT group was significantly limited compared to PBS group (24.5 ± 5.7% versus 36.0 ± 7.0%, *P* < 0.05).

On day 28 post MI surgery, Masson trichrome staining were applied to assess myocardial interstitial fibrosis (see in Figure 2B-2C) and our results illustrated that NPNT overexpression ameliorated myocardial interstitial fibrosis (11.4 ± 2.6% *vs.* 19.1 ± 1.6%, *P* < 0.05) compared with MI-PBS group, namely in border zone after MI.

A Kaplan-Meier survival analysis was performed up to 28 days post MI, as shown in Figure 2D. There were total 11 in 18 mice died in MI-PBS group, 12 in 18 mice in MI-EV group and 4 in 18 in MI-NPNT group. No mice died in the sham group, neither did the three MI group after 10 days post MI. The Kaplan-Meier survival curve revealed a significantly decreased mortality in the NPNT-overexpressing group compared with the MI-PBS group (*P* = 0.0197).

### NPNT promotes angiogenesis post myocardial infarction

**Figure 3:**
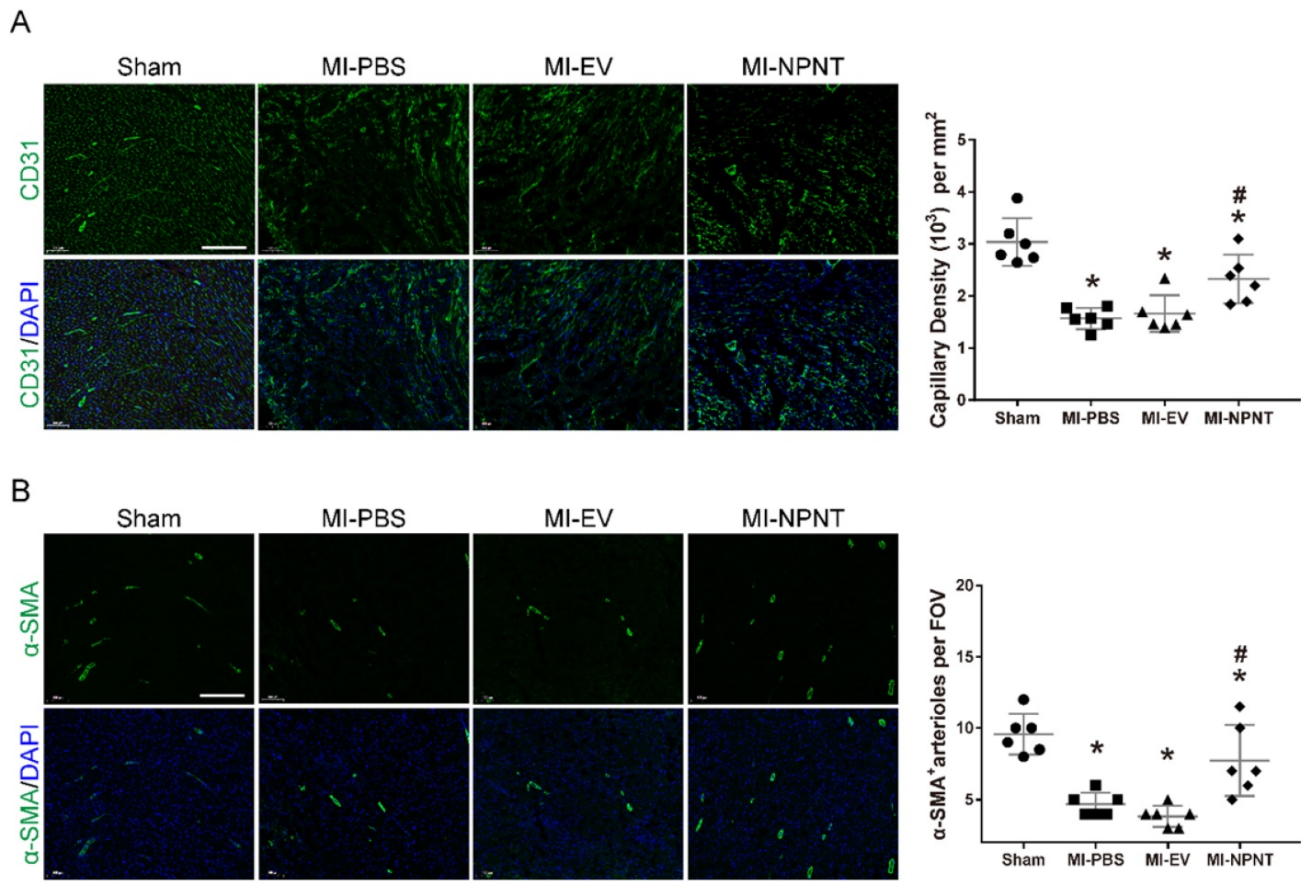
** NPNT promoted angiogenesis in infarcted mouse hearts. CD31 and α-SMA were immunofluorescent stained in infarct border zone at 4 weeks post-MI. (A)** Representative images and quantitative analysis of myocardial capillary density determined by the number of endothelial-specific CD31-positive cells per mm^2^ (n = 6).** (B)** Representative images and quantification of newly formed vessels analysed by α-SMA-positive arterioles per field of view (FOV) (n = 6). * *P* < 0.05 versus sham group. # *P* < 0.05 versus MI-PBS group. Scale bar, 200 µm.

As mentioned above, we observed a beneficial effect of NPNT on cardiac repair post MI. Insufficient post-MI myocardial angiogenesis has been regarded as a nonnegligible event which precipitates ventricular remodelling progression^24^. Paradigm that endothelial and smooth muscle cells interacting with each other to form new blood vessels has been widely accepted^25^. So next we would like to explore whether overexpression of NPNT influence the angiogenesis. Here we evaluated angiogenesis and arteriogenesis with sections (10 μm) from mouse hearts removed on day 28 post-MI using CD31 and a-SMA immunofluorescence staining, which are widely applied markers for quantification^26^, ^27^. As illustrated in Figure 3A, capillary density in the infarct border zone decreased dramatically post-MI operation. Then a significant increase in angiogenesis was observed in the MI-NPNT group as CD31 positive staining increased. Furthermore, a-SMA representing arteriogenesis was upregulated by NPNT in MI mouse hearts (Figure 3B). These results indicate that NPNT treatment improves angiogenesis and arteriogenesis *in vivo* after MI operation.

### NPNT promotes endothelial cell migration and tube formation

**Figure 4:**
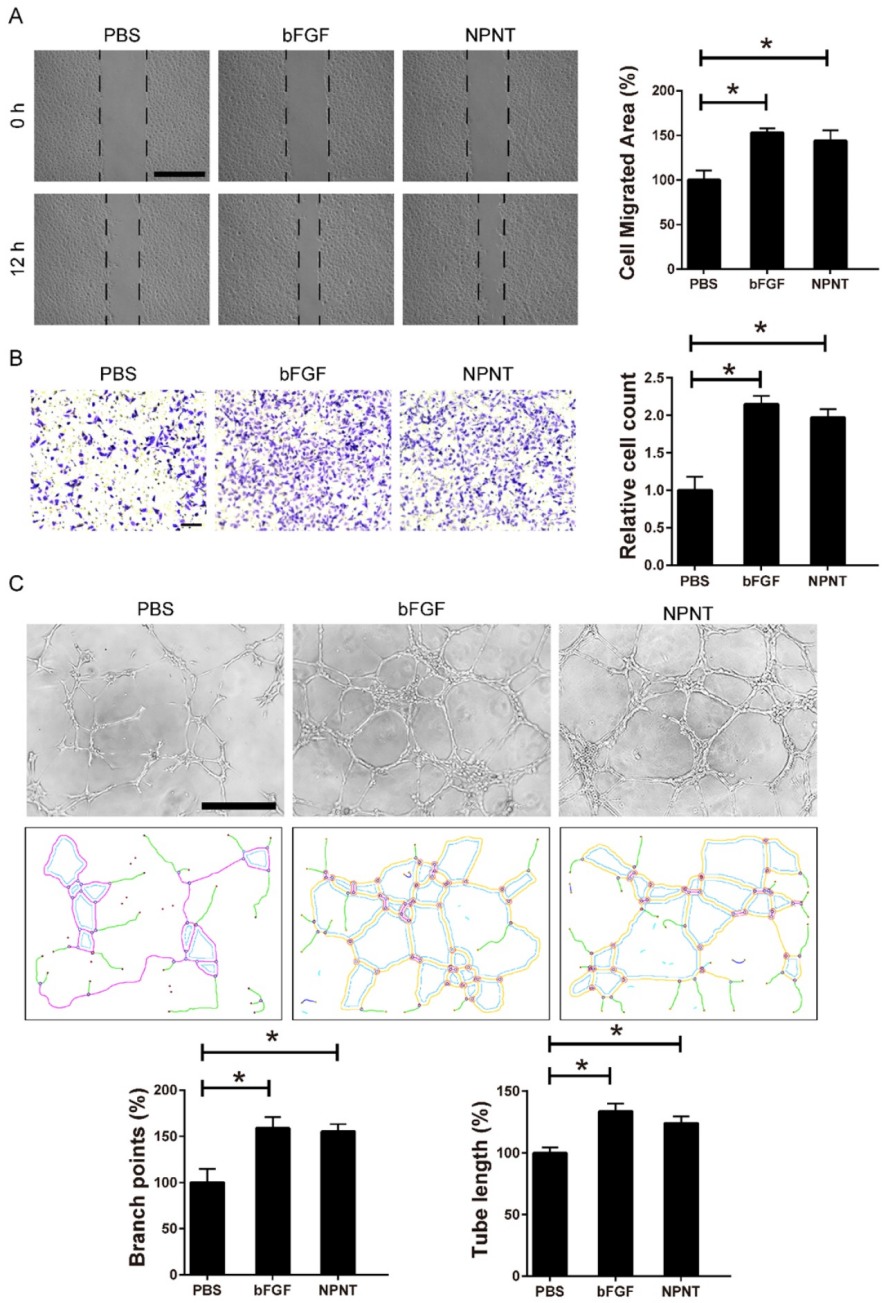
** NPNT induced endothelial cell wound healing and tube formation in vitro. (A)** Representative images and statistical analysis of the cell migration area of scratch wound healing assay in PBS, rm-NPNT (recombinant mouse NPNT, 500 ng/ml, same dose in following experiments unless indicated) and rh-bFGF (recombinant human bFGF, 50 ng/ml, same dose in following experiments unless indicated) treated HUVEC for 12h. Scale bar, 500 µm. **(B)** Representative images and quantification of migrated cells in transwell assay for 24h. Scale bar, 100 µm.** (C)** Representative images of tube-formation assay in HUVEC seeded on Matrigel (upper). Branching points and tube formatted were shown using Image J (lower). PBS, rm-NPNT and rh-bFGF was added into the culture medium, respectively. Quantification of branching points and tube length was calculated in 4 different fields. * *P* < 0.05. Scale bar, 500 µm.

Cell migration and tube formation are hallmarks of angiogenesis. Therefore, we performed migration assays and tube formation assays using primary HUVECs, a widely used cell line for endothelial cell biology. For wound healing assay, recombinant mouse NPNT protein (rmNPNT) was added immediately when the wound scratch was made. As illustrated in Figure 4A, rmNPNT significantly improved wound healing in HUVECs compared with the PBS group (negative control). For Transwell assay in Figure 4B, migrating HUVEC cell counts significantly increased if treated with NPNT compared with control group. Similarly, recombinant mouse NPNT protein stimulated HUVEC tube formation comparable with positive control (Figure 4C). Taken together, our results show that NPNT promotes endothelial cell migration and tube formation.

### NPNT promoted migration and tube formation of HUVECs through activating the EGFR/JAK2/STAT3 signalling pathway

Previous studies have indicated that initiating EGFR-mediated activation of a series of downstream signal factors could regulate angiogenesis^28^, ^29^. Therefore, we tested whether the effect of NPNT on HUVECs migration and tube formation was mediated through the EGFR-related signalling pathway or not. So we treated HUVECs with NPNT recombinant protein at different time points (0, 5, 10, 20, 30, 60 min) and collected total cell lysates for Western blot analysis.

**Figure 5:**
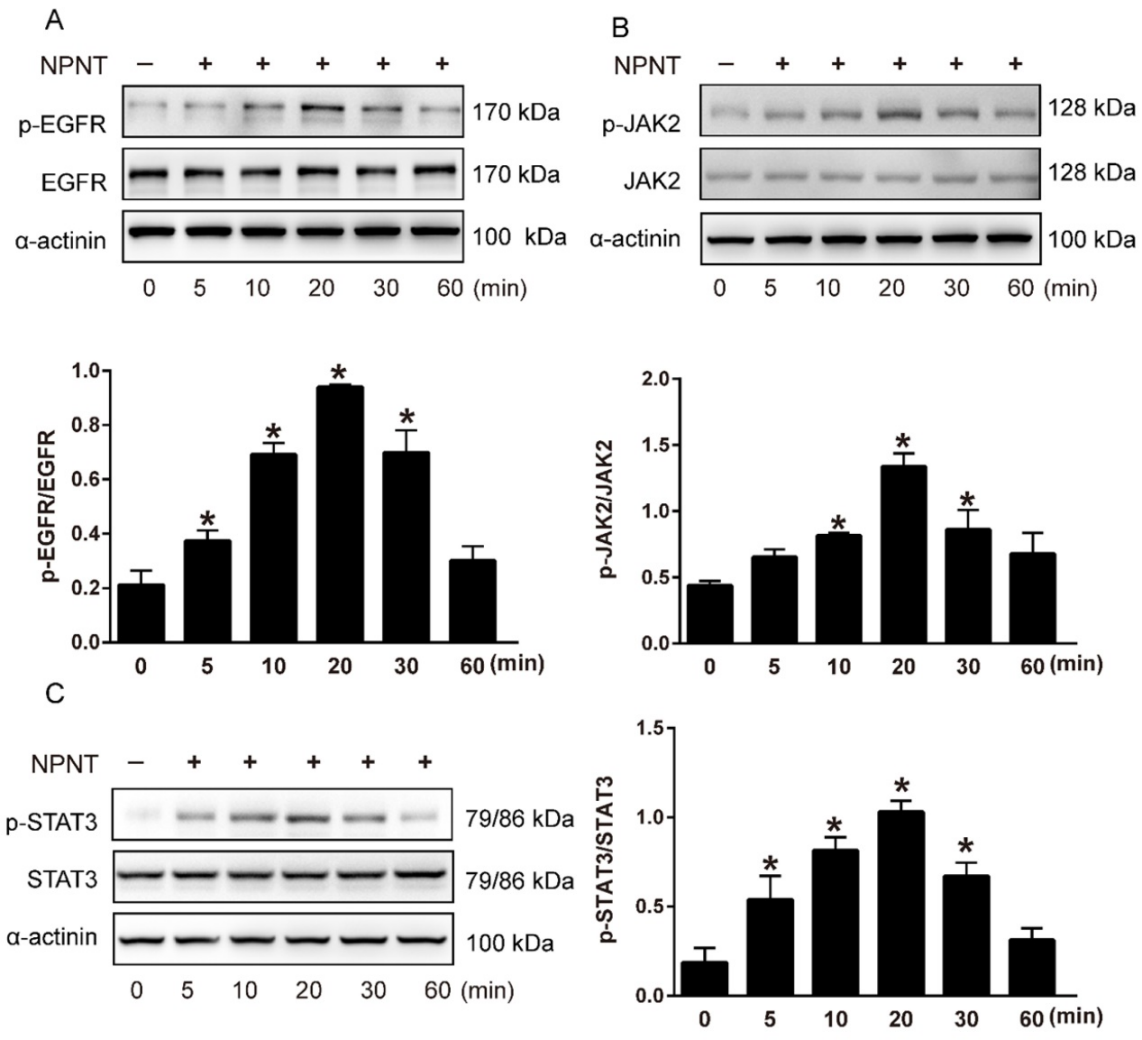
** NPNT activated the EGFR/JAK2/STAT3 signalling pathway in HUVECs. (A-C)** Recombinant mouse NPNT protein was added to HUVECs (500 ng/ml) over time (0 min, 5 min, 10 min, 20 min, 30 min, 60 min). The p-EGFR/EGFR ratio, p-JAK2/JAK2 ratio and p-STAT3/STAT3 were increased at 5 min, peaked at 20 min and decreased thereafter. Each bar represents the mean ± SD of three independent experiments. * *P* < 0.05 versus control group.

**Figure 6:**
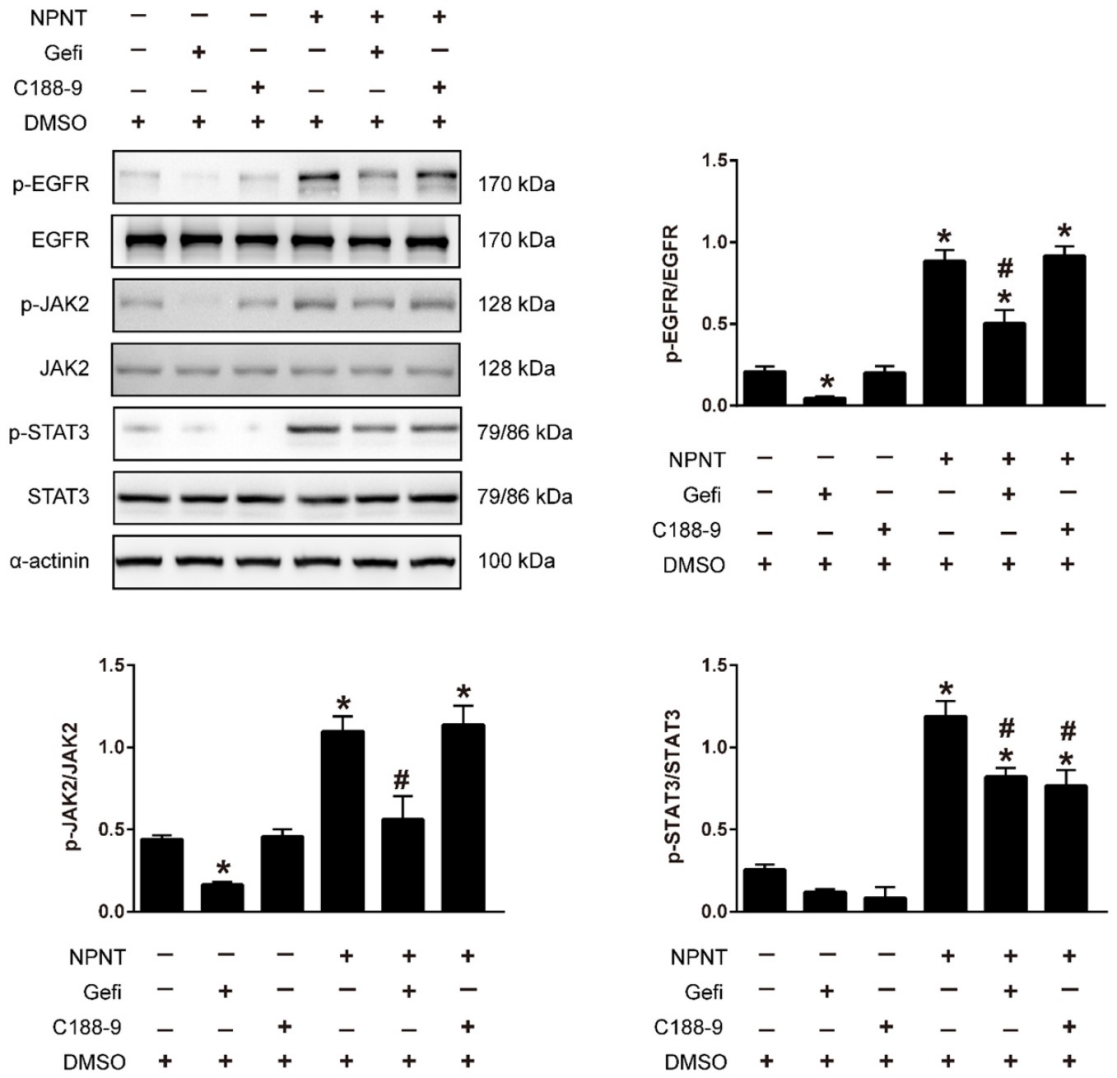
** Gefitinib and C188-9 abrogated NPNT-induced EGFR/JAK2/STAT3 signalling activation.** HUVECs were preincubated with Gefitinib (EGFR inhibitor, 10 μM) or C188-9 (STAT3 inhibitor, 10 μM) for 1 h and then stimulated with NPNT (500 ng/ml) for 20 min. The phosphorylation levels of EGFR, JAK2 and STAT3 were all decreased in Gefitinib pretreatment. While only phosphorylated STAT3 not phosphorylated EGFR or phosphorylated JAK2 was inhibited by C188-9 treatment, indicating NPNT activated EGFR followed by activating JAK2/STAT3 pathway. Gefitinib and C188-9 were dissolved with DMSO, thus identical dose of DMSO was added to the control group and NPNT group, respectively. * *P* < 0.05 versus DMSO group, # *P* < 0.05 versus NPNT group.

**Figure 7:**
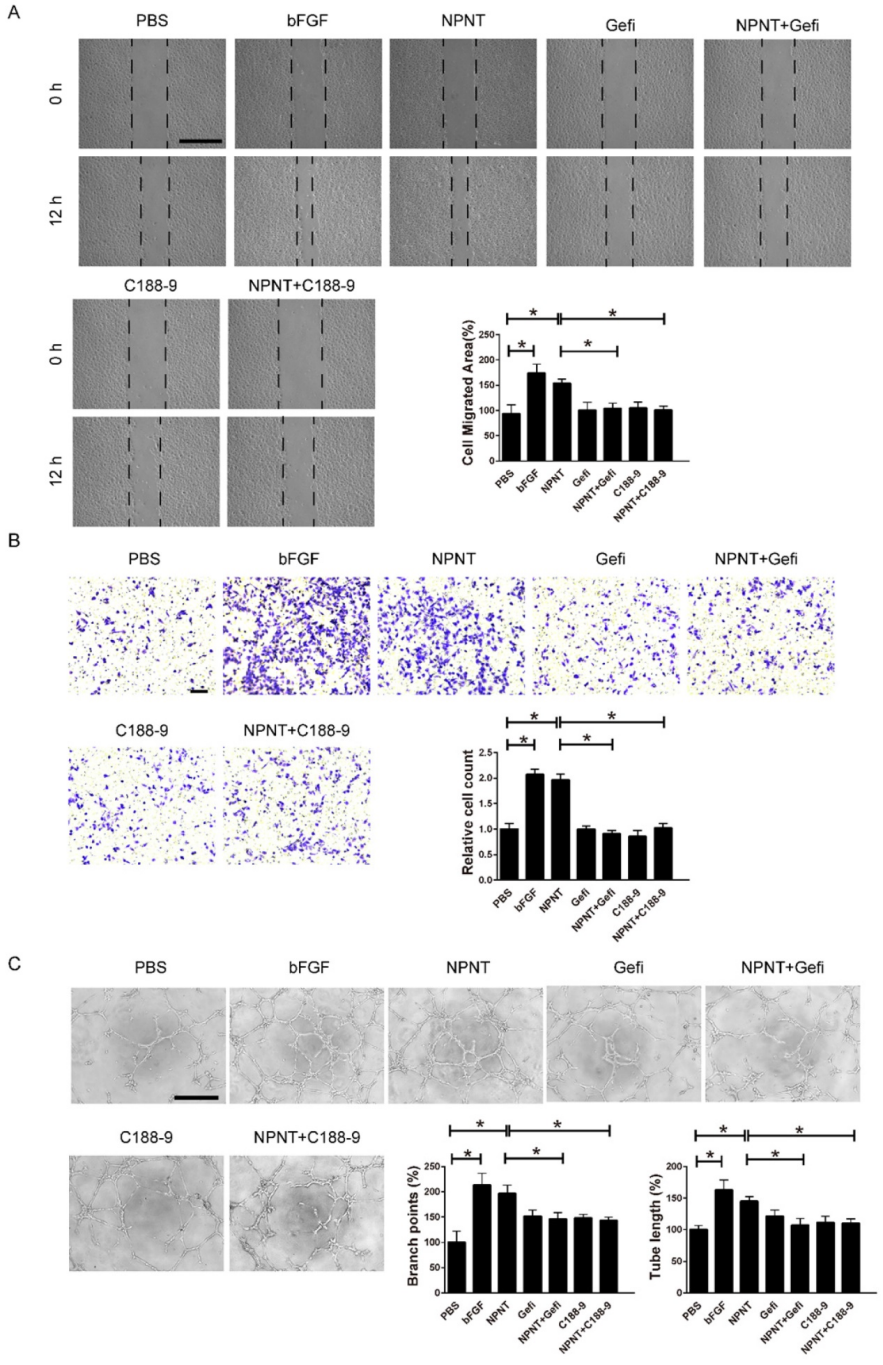
** Gefitinib and C188-9 impaired NPNT-induced endothelial cell migration and tube formation in vitro. (A)** Representative images and quantification of scratch wound healing assays (Scale bar, 500 μm),** (B)** Transwell assay (scale bar, 100 μm) and **(C)** tube formation assay (Scale bar, 500 μm) indicating that NPNT-promoted endothelial cell migration and tube formation were significantly inhibited by Gefitinib (10 μM) or C188-9 (10 μM). Quantitative analysis of NPNT-induced cell migration and tube formation. * *P* < 0.05.

**Figure 8:**
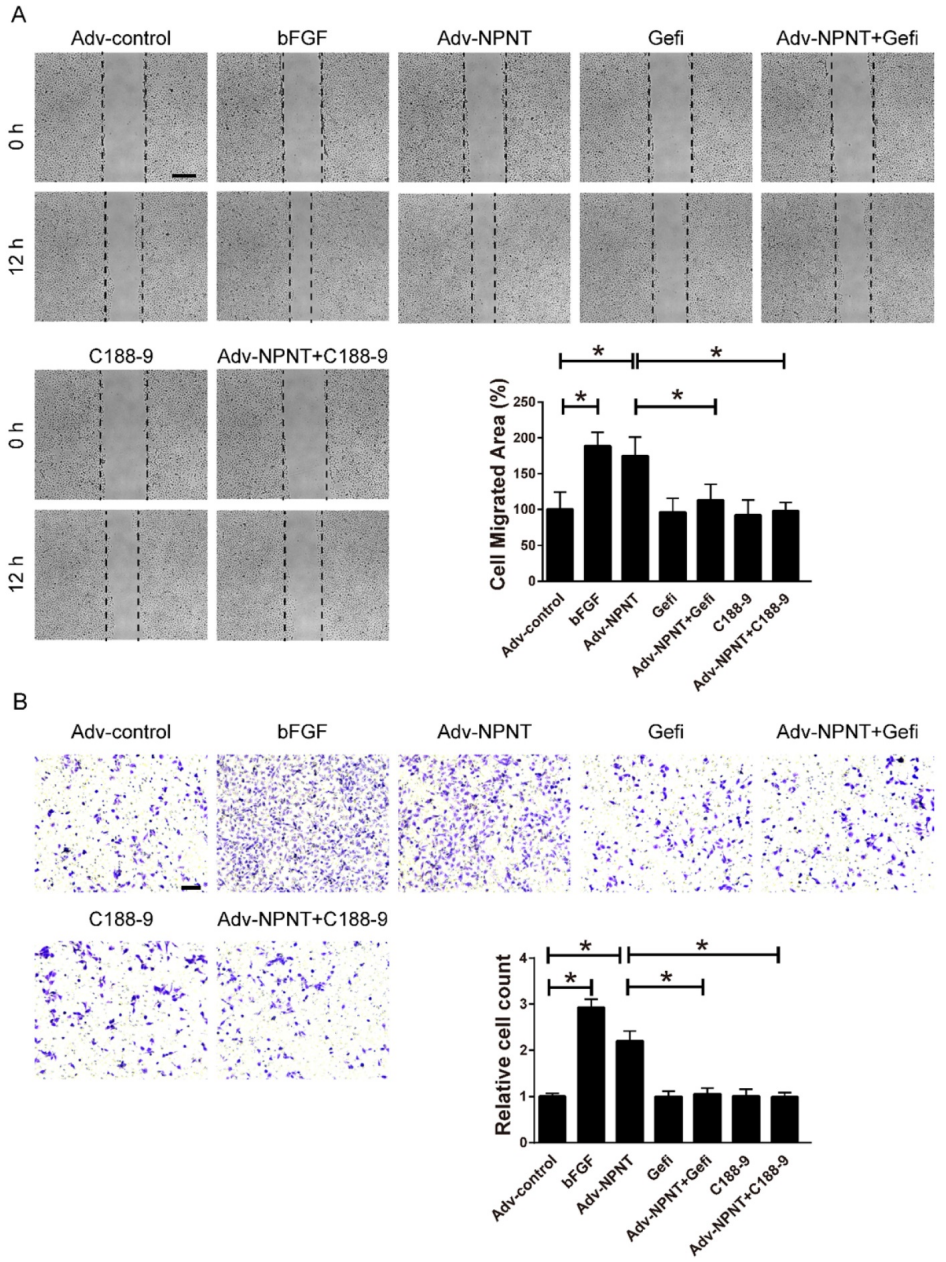
** Gefitinib and C188-9 impaired Adv-NPNT-induced endothelial cell migration in vitro. (A)** Representative images and quantification of scratch wound healing assays (Scale bar, 500 μm) and** (B)** Transwell assay indicating that Adv-NPNT-promoted endothelial cell migration and were significantly inhibited by Gefitinib (10 μM) or C188-9 (10 μM). * *P* < 0.05. Scale bar, 100 μm.

**Figure 9:**
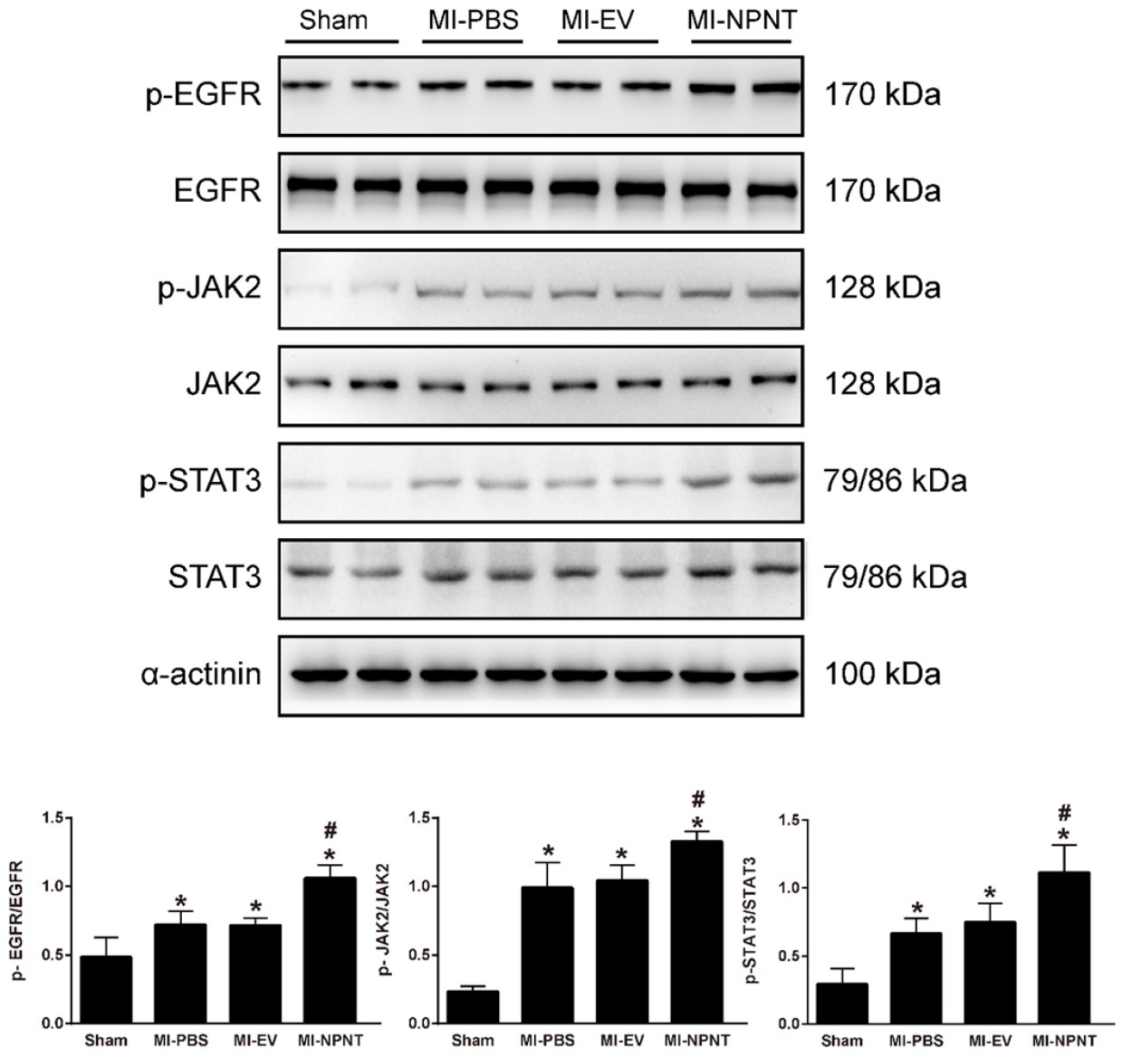
** NPNT activated EGFR/JAK2/STAT3 signalling pathway in vivo.** The phosphorylation levels of EGFR and its downstream factors JAK2, STAT3 were higher in the MI group mice hearts and further increased in the MI-NPNT group on day 28 post-MI. Each bar represents the mean ± SD of n = 4-6 mice in each group. * *P* < 0.05 versus sham group, #* P* < 0.05 versus MI-PBS group.

As shown in Figure 5A-5C, Western blot results indicated an increase for phosphorylated EGFR, which could be partly blocked by Gefitinib, an inhibitor of EGFR. In addition, activation of STAT3 has been implied during the process of angiogenesis^30^, and we further detected the JAK2/STAT3 signalling pathway, and found that NPNT led to the phosphorylation of JAK2 and STAT3, which could be easily blocked by C188-9, an inhibitor of STAT3 (Figure 6). Migration and tube formation assays also showed that the angiogenic capacity of NPNT was impaired by Gefitinib or C188-9 (Figure 7). In order to simulate virtual situation *in vivo*, we performed experiments in adult mouse cardiomyocytes (AMCM) and endothelial cells as followed. Adenovirus expressing mouse NPNT (pAd/CMV/V5-DEST -NPNT) infection induces expression of NPNT in AMCM (Supplementary Figure 3). Medium collected from AMCM with NPNT forced expression promotes the migration of endothelial cells (HUVEC and mBend3) (scratch and transwell experiments, Figure 8 and Supplementary Figure 4), which can be readily abrogated by Gefi and C188-9, further strengthening the conclusion that NPNT promotes migration and tube formation of HUVECs through activating the EGFR/JAK2/STAT3 signaling pathway. We further validify the activation of EGFR/JAK2/STAT3 signalling in mice myocardium using immunoblotting. As illustrated in Figure 9, the phosphorylation of EGFR and its downstream gene JAK2/STAT3 increased in the MI-NPNT group versus MI-PBS groups (Figure 9). Such findings indicate that NPNT promoted migration and tube formation of HUVECs potentially through activating the EGFR/JAK2/STAT3 signalling pathway, at least partially.

## Discussion

Current typical revascularization strategies such as percutaneous coronary intervention (PCI) and coronary artery bypass grafting (CABG) restore the macrovascularization in the acutely or chronically ischemic territory. Nevertheless, increasing evidence has demonstrated that many patients could also benefit from angiogenic approaches that would promote microvascularization during ischaemic repair^31^, ^32^. ECM proteins have been now widely demonstrated beneficial for angiogenesis. First recognized in mouse embryonic kidneys as a novel ligand for the integrin α8β1^33^, ^34^, NPNT is depicted by subsequent studies that is required in kidney and heart development^35^, ^36^. As a member of the epidermal growth factor (EGF)-like superfamily, NPNT protein contains five EGF-like repeat domains. Recently, it has been manifested that NPNT was strongly involved in promoting osteogenic angiogenesis *in vitro*^37^, which hints it might be a promising candidate molecule to enhance cardiac angiogenesis. However, the role of NPNT in the heart post MI remains enigmatic.

With respect to the function of NPNT for heart development and disorders, few previous studies have been performed to address this issue. A study in zebrafish identified NPNT as a pivotal regulator involved in atrioventricular canal differentiation via the Bmp4-Has2 signalling pathway^36^. In addition, recent studies found that NPNT contributes to cardiomyocyte adhesion, metabolic activity, contractility, cell-to-cell communication and cardiomyocyte cytokinesis 38, 39. There is transient expression of NPNT during rat heart development^36^, and a similar pattern was seen here, with a barely observed NPNT expression in adult mouse hearts. While in this study, we revealed that NPNT contributed to myocardial repair through modulating angiogenesis, highlighting the favourable function of NPNT for post MI injury.

Hitherto, NPNT has been reported to bind integrin with its RGD motif and EGFR with its EGF repeat domains successively^33^, ^40^. In this study, we also confirmed the physical interaction between NPNT and EGFR with either anti-EGFR neutralizing antibody or EGFR dominant negative plasmid. In each condition, the activation of EGFR/JAK/STAT3 signaling could be abrogated in HUVEC (Supplementary Figure 5). It has been widely accepted that signalling through EGFR regulates pro-angiogenic factors in various cells^41^, ^42^. Our data emphasis the essential role of EGFR signalling for NPNT working. EGFR signalling involved in angiogenesis has been reported through several downstream pathways, such as PI3K/AKT/ERK. Actually, MAPK signalling has been reported triggered by NPNT by binding to EGFR via its EGF-repeat domain^40^. However, we observed no significant phosphorylation for AKT in heart overexpressing NPNT (data now shown), while the phosphorylation of EGFR and downstream JAK2/STAT3 were all upregulated in the MI-NPNT group when compared with the MI-PBS and the MI-EV group. In addition, NPNT-induced EGFR activation in cell experiment results could be obviated by an EGFR or STAT3 inhibitor, verifying that the EGFR/JAK2/STAT3 signalling pathway plays a vital role in NPNT-induced angiogenesis. Thus, we concluded the possible mechanism of NPNT in angiogenesis using a schematic diagram as shown in the Graphical abstract.

Although our results are encouraging, the current study had some limitations. First, there might be other mechanisms involved in this process; nevertheless, we have identified one of the possible mechanisms. Second, the present study contained no human specimens or treatments; thus, further work is required to draw a conclusion regarding humans. Third, this study did not observe the impact of EGFR or STAT3 inhibitors in vivo model in the presence of NPNT. Please note that angiogenesis usually refers to arterial system rather than venous system, our current results are based on HUVECs, future studies are warranted using arterial endothelial cells to further explore the role of nephronectin on angiogenesis *in vitro*.

Collectively, the present study found that NPNT promoted angiogenesis and improved cardiac function in infarcted mouse hearts through the EGFR/JAK2/STAT3 signalling pathway. Our study might provoke future research on NPNT and cardiac repair.

## Conclusion

Here, we identified NPNT as an angiogenesis modulator during myocardial repair, and provided evidence that nephronectin limits infarct size and benefits survival by promoting angiogenesis, arteriogenesis, and alleviating cardiac fibrosis in experimental myocardial infarction, probably through promoting endothelial cell migration and tube formation. Our study further revealed that the proangiogenic effect of NPNT may be driven by activating the EGFR/JAK2/STAT3 signalling pathway.

## Supplementary Material

Supplementary figures.
