# Supplementary material for: Nephronectin promotes cardiac repair post myocardial infarction via activating EGFR/JAK2/STAT3 pathway
Source: Int J Med Sci. 2022 May 13;19(5):878–92. doi: 10.7150/ijms.71780 (PMC9149649; doi:10.7150/ijms.71780)
Supplement: Supplementary file 1 — Supplementary figures. [file ijmsv19p0878s1.pdf]

Supplementary Material

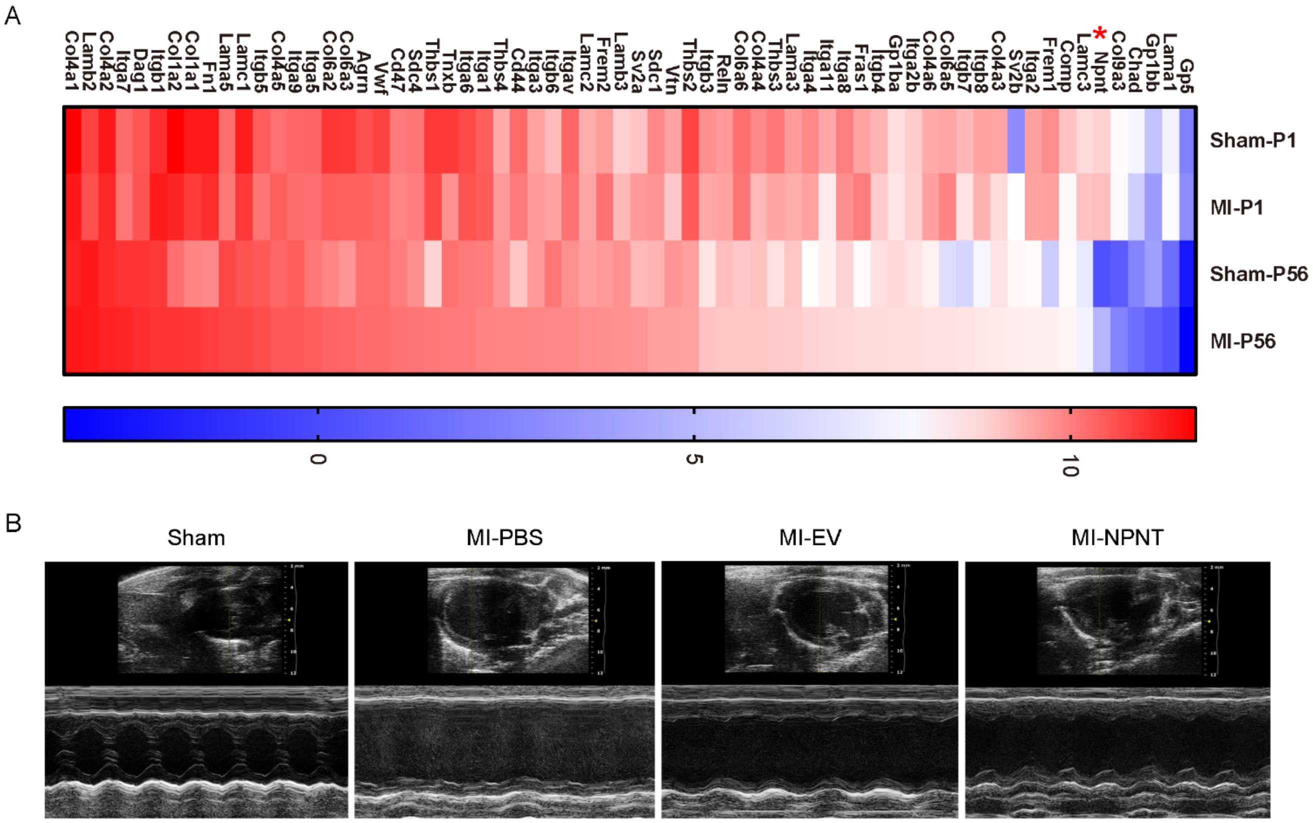

Supplementary Figure 1. NPNT plays a role in cardiac development and during MI insult. (A)Heatmap focused on ECM protein genes reassembled based on open access RNA-seq data from Gene Expression Omnibus under the accession number GSE95755. (B) Representative images of standard M-mode echocardiography performed on day 28 post MI in the Sham group, MI-PBS group, MI-EV group and MI-NPNT group.

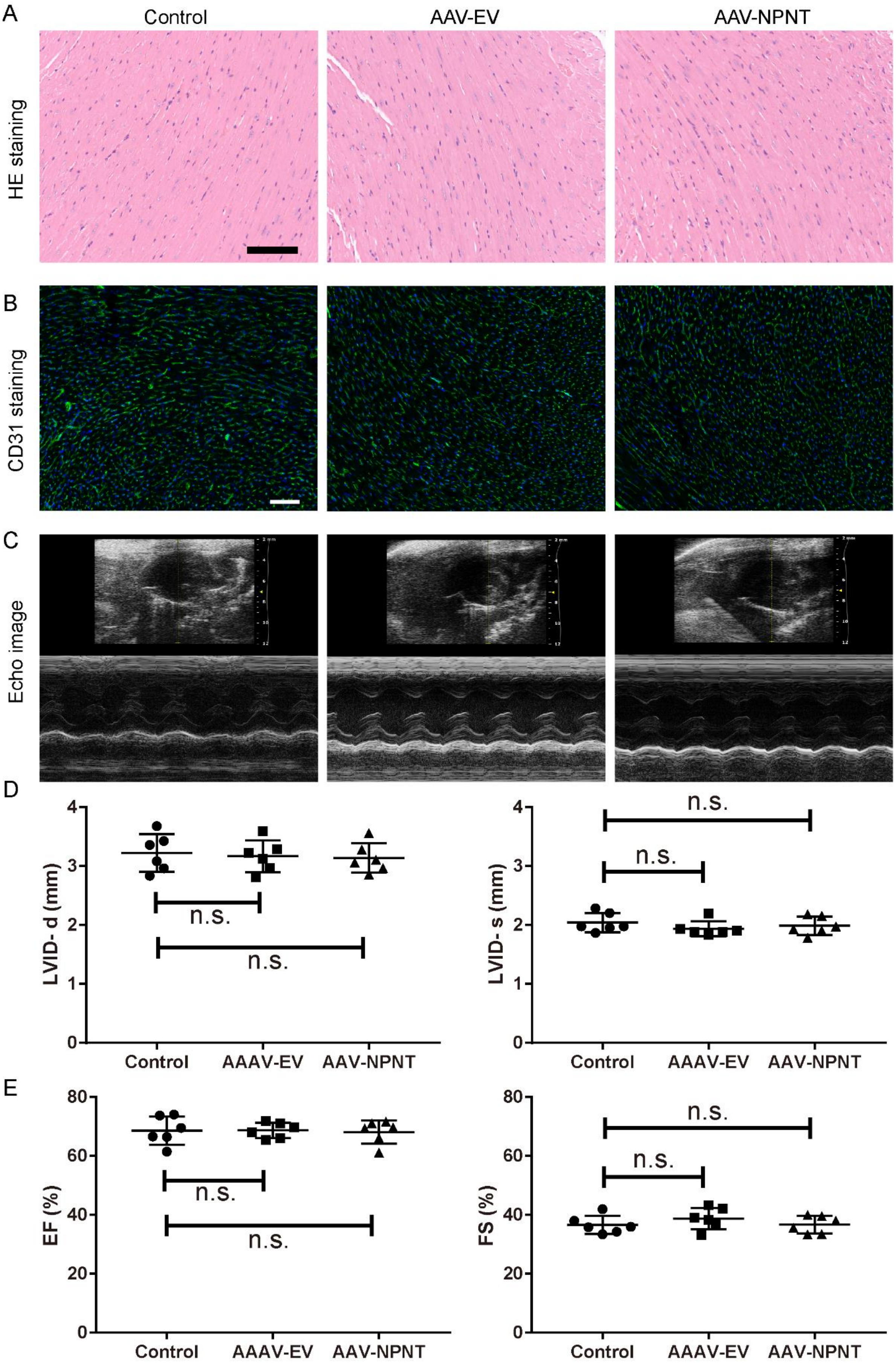
A
Control
AAV-EV
AAV-NPNT
HE staining
B
CD31 staining
C
Echo image
D
LVID- d (mm)
LVID- s (mm)
n.s.
Control
AAAV-EV
AAV-NPNT
E
EF (%)
FS (%)
n.s.
Control
AAAV-EV
AAV-NPNT

Supplementary Figure 2. NPNT gene overexpression by adeno-associated virus (serotype 2/9) in mouse hearts did not affect myocardial morphology, capillary density, or cardiac function. Three weeks after the AAV injection, several mice from the MI-EV, MI-NPNT group and control group (n= 3-6 for each group) were sacrificed and subjected to (A) HE staining, scale bar, 200 µm; (B) CD31staining, Scale bar, 100 µm; (C-E) echocardiography to explore myocardial morphology, capillary density, and cardiac function under the premise of overexpression of NPNT in hearts.

A

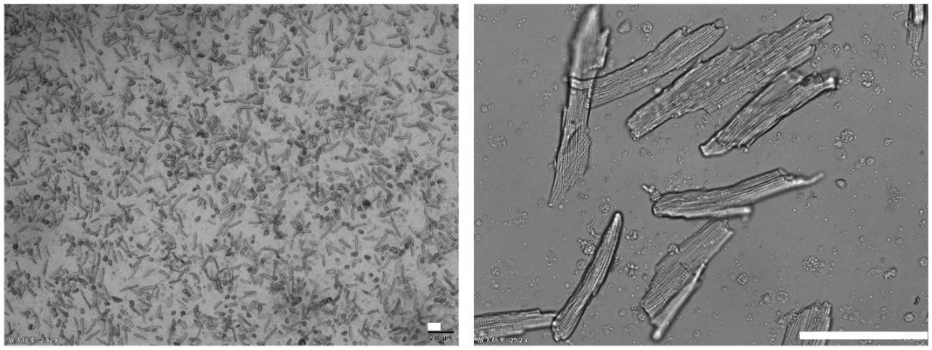

B

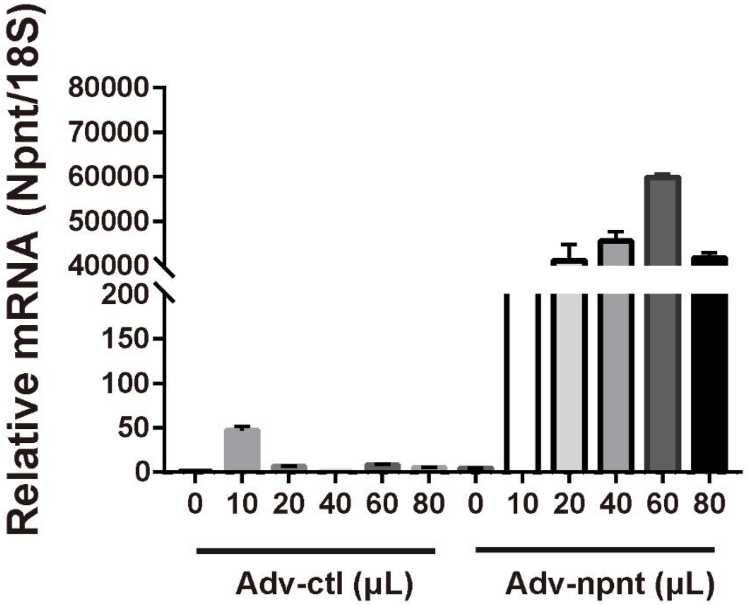

Supplementary Figure 3. Adult mouse cardiomyocytes (AMCM) expressing mouse NPNT via being infected by pAd/CMV/V5-DEST-NPNT. A. Adult mouse cardiomyocytes (AMCM) representative images with different magnifications in brightfield (these two images come from two experiments). Scale bar, 100 µm; B. qPCR results showed pAd/CMV/V5-DEST-NPNT infected AMCM in different virus titer. Sixty µl/mL virus titer was used in the later experiments.

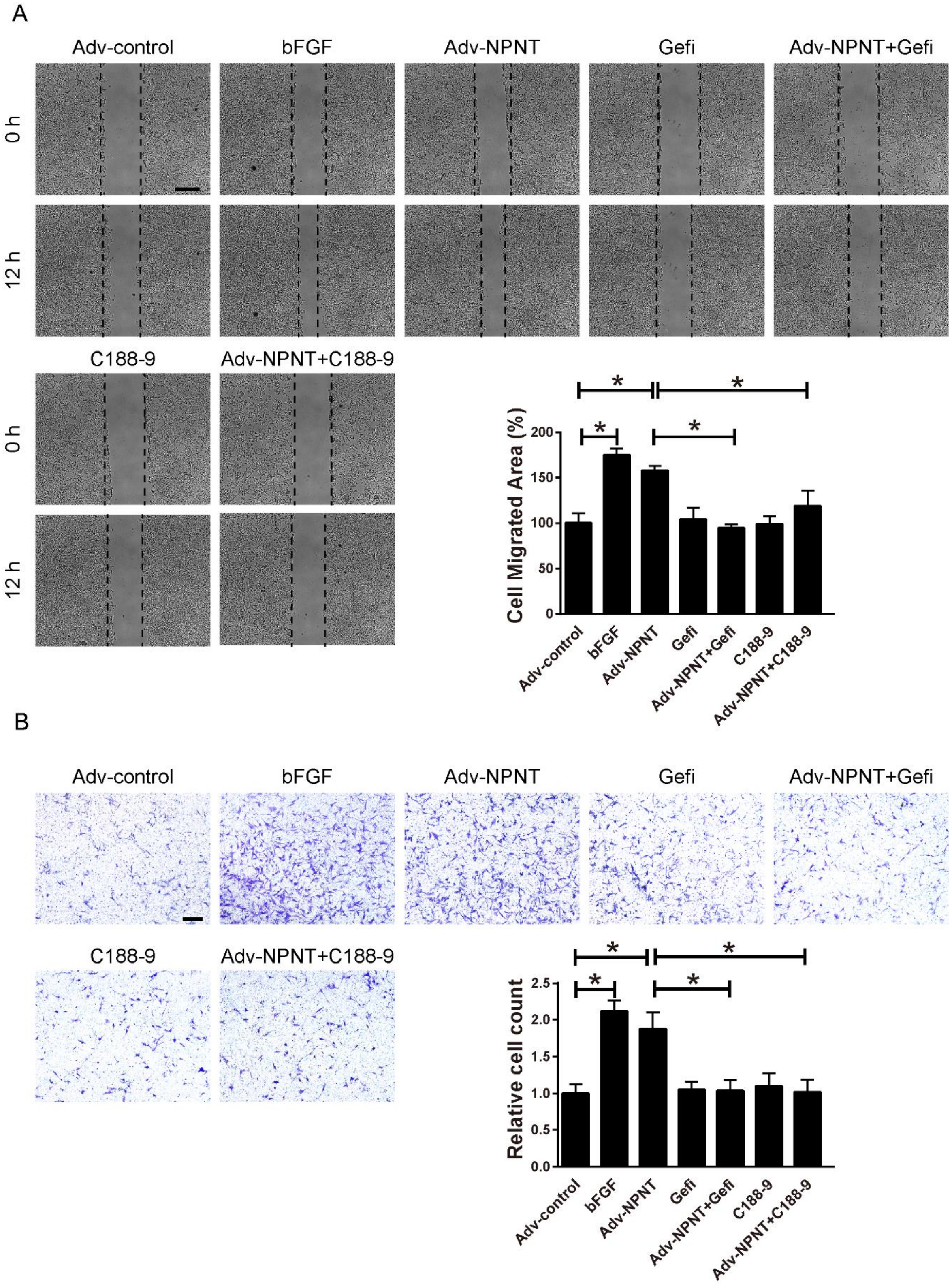

Supplementary Figure 4. Gefitinib and C188-9 impaired Adv-NPNT-induced mouse endothelial cell migration in vitro. (A) Representative images and quantification of scratch wound healing assays, (B) Transwell assay indicating that Adv-NPNT-promoted endothelial cell migration and were

significantly inhibited by Gefitinib (10 μM) or C188-9 (10 μM). Quantitative analysis of NPNT-induced cell migration. * $P < 0.05$. Scale bar, 100 μm.

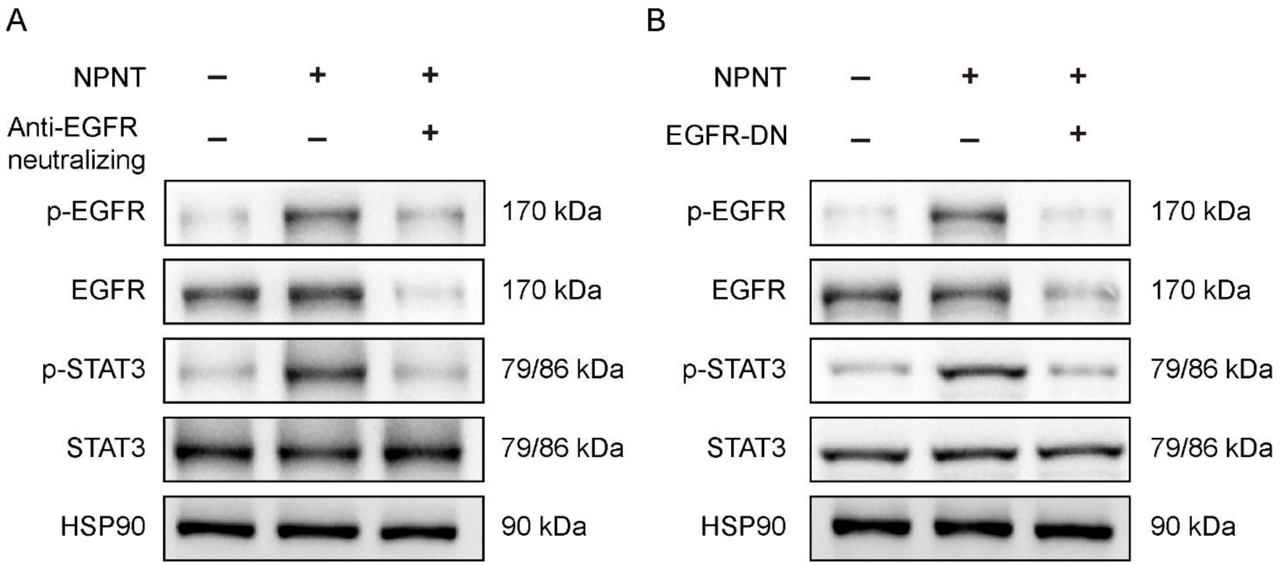

Supplementary Figure 5. Anti-EGFR neutralizing antibody and EGFR dominant negative plasmid abrogated EGFR/JAK/STAT3 activation by NPNT. A. Anti-EGFR neutralizing antibody was added to culture medium 1 hour before NPNT stimulation and then EGFR/JAK/STAT3 pathway was detected. B. EGFR dominant negative plasmid was transfected in HUVEC for 24 hours before NPNT stimulation and then EGFR/JAK/STAT3 pathway was detected.
